# Supplementary material for: SC-Track: a robust cell-tracking algorithm for generating accurate single-cell lineages from diverse cell segmentations
Source: Brief Bioinform. 2024 Apr 27;25(3):bbae192. doi: 10.1093/bib/bbae192 (PMC11070058; doi:10.1093/bib/bbae192)
Supplement: Supplementary_Figures_FINAL_bbae192 [file supplementary_figures_final_bbae192.docx]

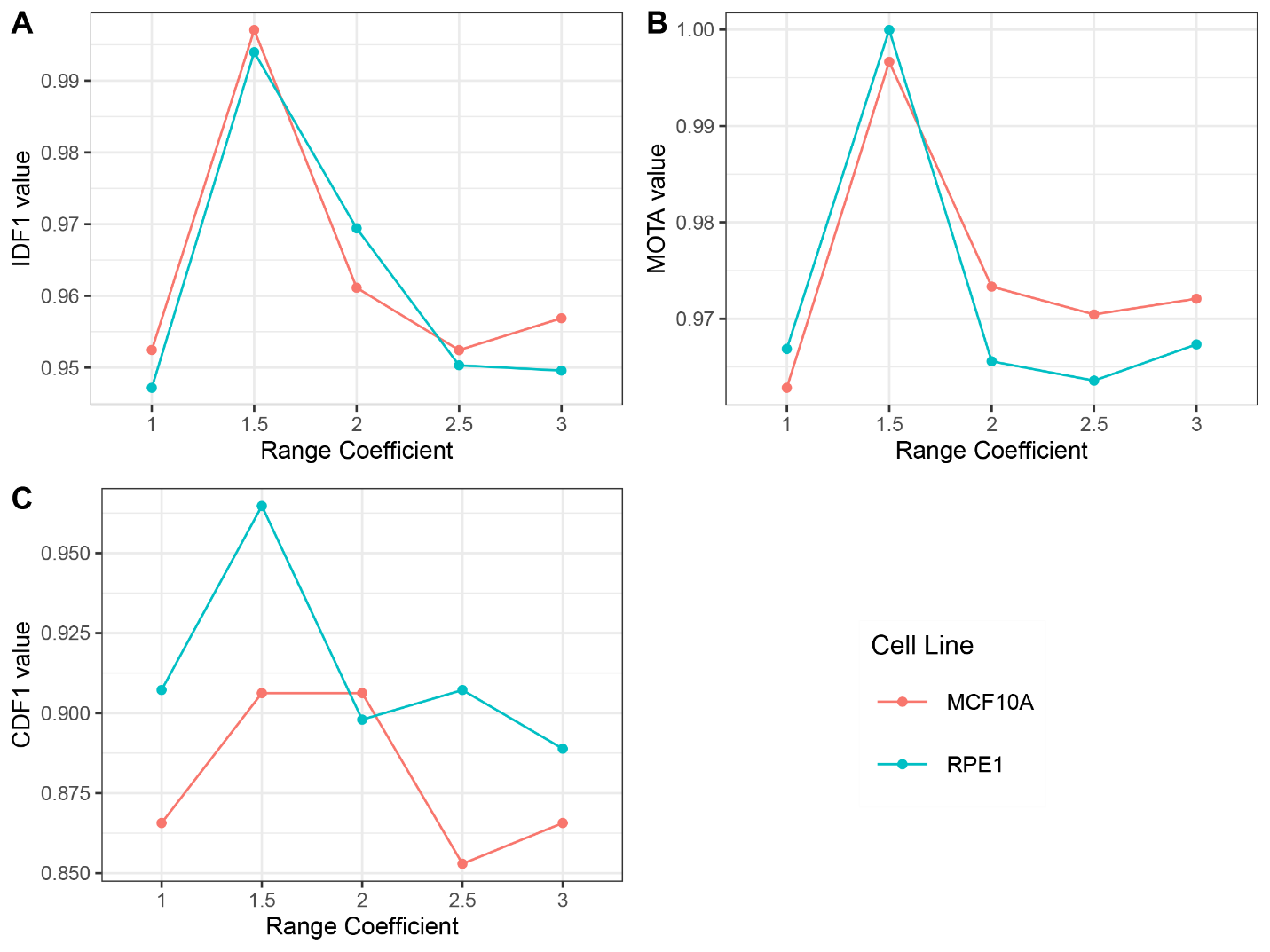


***Fig. S1: The effect of changing the “available_range_coefficient” parameter, which decides the degree of bounding box search area expansion relative to the nucleus size of the unlinked segmented cell during tracking.***

Line plots showing the respective (A) IDF1, (B) MOTA and (C) CDF1 scores when the range coefficient is changed.


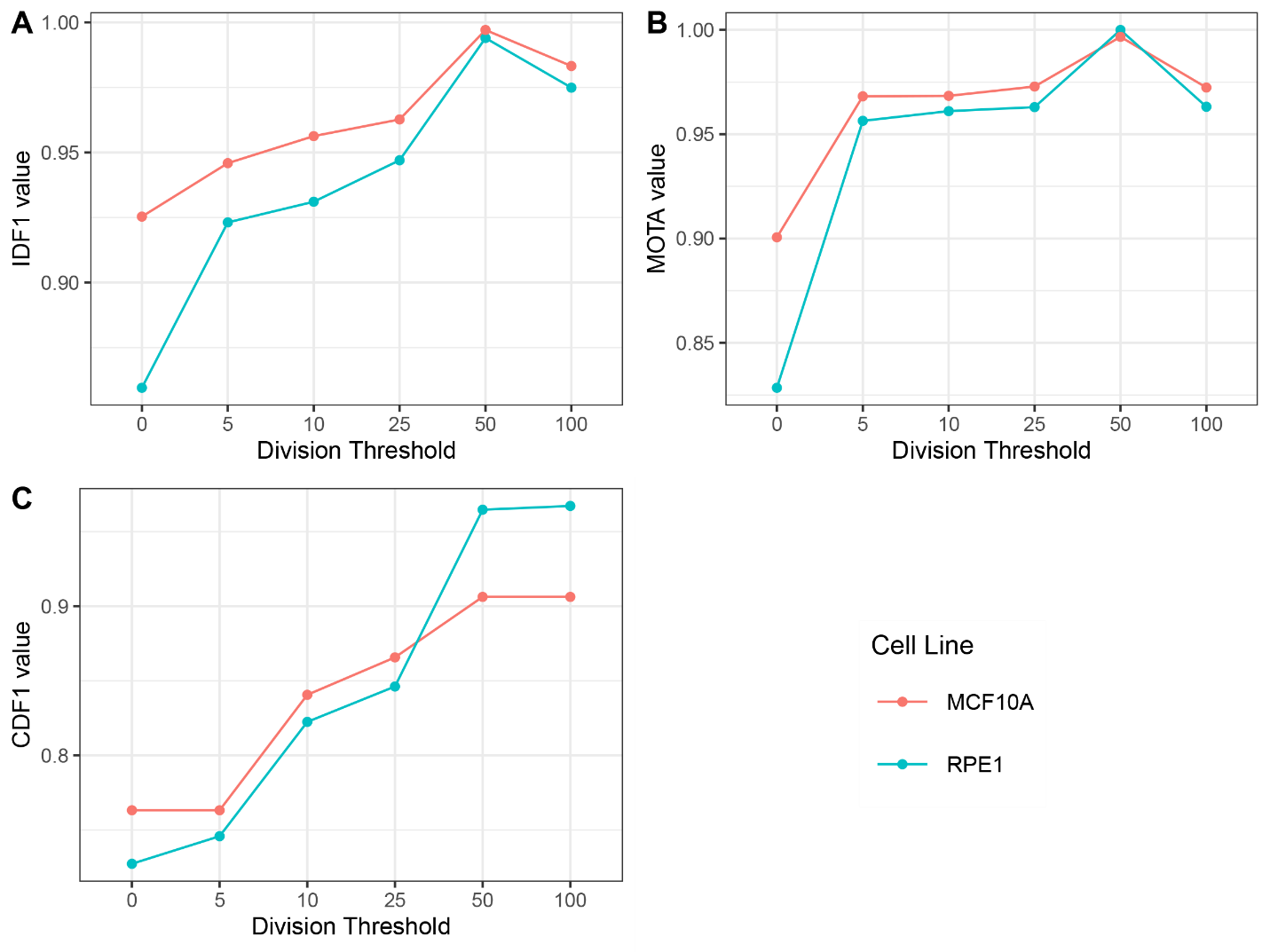


***Fig. S2: The effect of changing the “enter_division_threshold”, which represents the minimum number of frames before a candidate mother cell TrackTree can undergo a cell division event again.***

Line plots showing the respective (A) IDF1, (B) MOTA and (C) CDF1 scores when the division threshold is changed.
