# Supplementary material for: SC-Track: a robust cell-tracking algorithm for generating accurate single-cell lineages from diverse cell segmentations
Source: Brief Bioinform. 2024 Apr 27;25(3):bbae192. doi: 10.1093/bib/bbae192 (PMC11070058; doi:10.1093/bib/bbae192)
Supplement: Supplementary_tables_bbae192 [file supplementary_tables_bbae192.docx]

Supplementary Table 1: Development dataset used in the development and finetuning of SC-Track tracking parameters.

| Dataset | Cell Type | Number of image frames | Total Cells | Average cells per frame | Temporal resolution (minutes) |
| --- | --- | --- | --- | --- | --- |
| sub-rpe-1 | RPE1 | 100 | 5374 | 53.74 | 5 |
| sub-rpe-2 | RPE1 | 100 | 6056 | 60.56 | 5 |
| sub-rpe-3 | RPE1 | 100 | 7296 | 72.96 | 5 |
| sub-rpe-4 | RPE1 | 69 | 5673 | 82.22 | 5 |

Supplementary Table 2: SC-Track evaluation dataset summary.

| Dataset | Original filename | Cell Type | Number of image frames | Average cells per frame | Temporal resolution (minutes) | G1/G2 | S | M | Cell division events |
| --- | --- | --- | --- | --- | --- | --- | --- | --- | --- |
| RPE1-01 | copy_of_1_xy01 | RPE1 | 369 | 63.41 | 5 | 20760 | 2082 | 552 | 44 |
| RPE1-02 | copy_of_1_xy19 | RPE1 | 369 | 66.16 | 5 | 22624 | 1202 | 567 | 32 |
| MCF10A-01 | MCF10A_copy02 | MCF10A | 101 | 119.89 | 5 | 11147 | 541 | 345 | 29 |
| MCF10A-02 | MCF10A_copy11 | MCF10A | 101 | 70.75 | 5 | 6236 | 572 | 211 | 17 |
| RPE1-03 | src06 | RPE1 | 577 | 34.50 | 5 | 18459 | 1395 | 114 | 11 |

Supplementary Table 3: SC-Track evaluation dataset with uncorrected raw segmentations and classifications.

| Dataset | Detected instances | | | Cell classifications | | | G1/G2 phase classification | | | S phase classification | | | Mitosis phase classification | | |
| --- | --- | --- | --- | --- | --- | --- | --- | --- | --- | --- | --- | --- | --- | --- | --- |
|  | TP | FP | FN | TP | FP | FN | TP | FP | FN | TP | FP | FN | TP | FP | FN |
| RPE1-01 | 23121 | 245 | 279 | 21742 | 1624 | 1658 | 19474 | 493 | 1321 | 1935 | 952 | 172 | 333 | 179 | 165 |
| RPE1-02 | 24031 | 499 | 382 | 22088 | 2442 | 2325 | 19145 | 1495 | 1190 | 2342 | 641 | 711 | 601 | 306 | 424 |
| MCF10A-01 | 11418 | 324 | 691 | 11088 | 654 | 1021 | 10008 | 418 | 756 | 562 | 35 | 124 | 518 | 201 | 141 |
| MCF10A-02 | 6812 | 120 | 334 | 5549 | 1383 | 1597 | 4818 | 414 | 1199 | 588 | 782 | 125 | 143 | 187 | 273 |
| RPE1-03 | 19651 | 158 | 255 | 18724 | 1085 | 1182 | 17392 | 366 | 916 | 1282 | 455 | 206 | 50 | 264 | 60 |

Supplementary Table 4: SC-Track synthetic evaluation dataset with randomly deleted segmented images.

| Dataset | Detection loss ratio (%) | Average cells per frame | Detected cells per frame | G1/G2 phase classification | | | S phase classification | | | M phase classification | | |
| --- | --- | --- | --- | --- | --- | --- | --- | --- | --- | --- | --- | --- |
|  |  |  |  | TP | FP | FN | TP | FP | FN | TP | FP | FN |
| RPE1-01 | 5 | 63.41463 | 59.6802 | 19569 | 0 | 1226 | 1978 | 0 | 129 | 475 | 0 | 23 |
|  | 10 | 63.41463 | 56.523 | 18535 | 0 | 2260 | 1873 | 0 | 234 | 449 | 0 | 49 |
|  | 20 | 63.41463 | 50.3008 | 16491 | 0 | 4304 | 1680 | 0 | 427 | 390 | 0 | 108 |
|  | 30 | 63.41463 | 43.9756 | 14424 | 0 | 6371 | 1458 | 0 | 649 | 345 | 0 | 153 |
|  | 50 | 63.41463 | 31.4607 | 10317 | 0 | 10478 | 1043 | 0 | 1064 | 249 | 0 | 249 |
| RPE1-02 | 5 | 66.15989 | 62.4526 | 19167 | 0 | 1168 | 2905 | 0 | 148 | 972 | 0 | 53 |
|  | 10 | 66.15989 | 59.1247 | 18176 | 0 | 2159 | 2734 | 0 | 319 | 906 | 0 | 119 |
|  | 20 | 66.15989 | 52.5474 | 16180 | 0 | 4155 | 2412 | 0 | 641 | 797 | 0 | 228 |
|  | 30 | 66.15989 | 45.8753 | 14096 | 0 | 6239 | 2126 | 0 | 927 | 705 | 0 | 320 |
|  | 50 | 66.15989 | 32.8482 | 10099 | 0 | 10236 | 1557 | 0 | 1496 | 464 | 0 | 561 |
| MCF10A-01 | 5 | 119.8911 | 113.4455 | 10187 | 0 | 577 | 648 | 0 | 38 | 623 | 0 | 36 |
|  | 10 | 119.8911 | 107.4356 | 9644 | 0 | 1120 | 611 | 0 | 75 | 596 | 0 | 63 |
|  | 20 | 119.8911 | 95.5743 | 8574 | 0 | 2190 | 559 | 0 | 127 | 520 | 0 | 139 |
|  | 30 | 119.8911 | 83.4554 | 7487 | 0 | 3277 | 471 | 0 | 215 | 471 | 0 | 188 |
|  | 50 | 119.8911 | 59.6535 | 5355 | 0 | 5409 | 342 | 0 | 344 | 328 | 0 | 331 |
| MCF10A-02 | 5 | 70.75248 | 66.6139 | 5662 | 0 | 355 | 676 | 0 | 37 | 390 | 0 | 26 |
|  | 10 | 70.75248 | 63.1881 | 5370 | 0 | 647 | 635 | 0 | 78 | 377 | 0 | 39 |
|  | 20 | 70.75248 | 56.198 | 4754 | 0 | 1263 | 572 | 0 | 141 | 350 | 0 | 66 |
|  | 30 | 70.7525 | 49.0495 | 4169 | 0 | 1848 | 491 | 0 | 222 | 294 | 0 | 122 |
|  | 50 | 70.7525 | 35.1584 | 2974 | 0 | 3043 | 378 | 0 | 335 | 199 | 0 | 217 |
| RPE1-03 | 5 | 34.4991 | 32.4974 | 17243 | 0 | 1065 | 1406 | 0 | 82 | 102 | 0 | 8 |
|  | 10 | 34.4991 | 30.5546 | 16232 | 0 | 2076 | 1304 | 0 | 184 | 94 | 0 | 16 |
|  | 20 | 34.4991 | 27.2704 | 14503 | 0 | 3805 | 1146 | 0 | 342 | 86 | 0 | 24 |
|  | 30 | 34.4991 | 23.6118 | 12536 | 0 | 5772 | 1017 | 0 | 471 | 71 | 0 | 39 |
|  | 50 | 34.4991 | 16.9636 | 9005 | 0 | 9303 | 731 | 0 | 757 | 52 | 0 | 58 |

Supplementary Table 5: Cell Tracking Challenge Silver Reference datasets.

| Dataset | Cell Type | Number of frames | Image Size (pixel, width x height) | Total Cells | Average cells per frame | Cell division events | Temporal resolution (minutes) | Image Type | Mask Type | Microscope | Microscope Objective |
| --- | --- | --- | --- | --- | --- | --- | --- | --- | --- | --- | --- |
| DIC-C2DH-HeLa | HeLa | 24 | 512x512 | 279 | 11.63 | 2 | 10 | DIC | Cell Mask | Zeiss LSM 510 Meta | Plan-Apochromat 63x/1.4 (oil) |
| PhC-C2DH-U373 | Glioblastoma-astrocytoma U373 | 115 | 696x520 | 880 | 7.65 | 0 | 15 | Phase Contrast | Cell Mask | Nikon | Plan Fluor DLL 20x/0.5 |
| Fluo-C2DL-MSC | Rat mesenchymal stem cell | 48 | 992x832 | 475 | 9.90 | 1 | 20 | Fluorescence | Cell Mask | PerkinElmer UltraVIEW ERS | Plan-Neofluar 10x/0.3 (Plan-Apo 20x/0.75) |
| Fluo-N2DH-GOWT1 | GFP-GOWT1 mouse stem cell | 92 | 1024x1024 | 2146 | 23.33 | 1 | 5 | Fluorescence | Nuclear Mask | Leica TCS SP5 | Plan-Apochromat 63x/1.4 (oil) |
| Fluo-N2DH-SIM+ | Simulated nuclei of HL60 cell | 100 | 773x739 | 1642 | 16.42 | 16 | 29 | Fluorescence | Nuclear Mask | Zeiss Axiovert 100S with a Micromax 1300-YHS camera | Plan-Apochromat 40x/1.3 (oil) |
| PhC-C2DL-PSC | Pancreatic stem cell | 100 | 576x720 | 9208 | 92.08 | 44 | 10 | Phase Contrast | Cell Mask | Olympus ix-81 | UPLFLN 4XPH |
| Fluo-N2DL-HeLa | HeLa | 92 | 700x110 | 6894 | 94.50 | 0 | 30 | Fluorescence | Nuclear Mask | Olympus IX81 | Plan 10x/0.4 |
| BF-C2DL-MuSC | Mouse muscle stem cell | 1100 | 1036x1070 | 2594 | 2.36 | 5 | 5 | Bright Field | Cell Mask | Zeiss PALM/ AxioObserver Z1 | EC Plan-Neofluar 10x/0.30 Ph1 |
